# A peripheral subepithelial network for chemotactile processing in the predatory sea slug *Pleurobranchaea californica*

**DOI:** 10.1371/journal.pone.0296872

**Published:** 2024-02-08

**Authors:** Tigran Norekian, Yichen Liu, Ekaterina D. Gribkova, Jilai Cui, Rhanor Gillette

**Affiliations:** 1 Whitney Laboratory for Marine Biosciences, University of Florida, St. Augustine, Florida, United States of America; 2 Friday Harbor Laboratories, University of Washington, Friday Harbor, Washington, United States of America; 3 Department of Molecular & Integrative Physiology, University of Illinois at Urbana-Champaign, Urbana, Illinois, United States of America; 4 Coordinated Science Laboratory, University of Illinois at Urbana-Champaign, Urbana, Illinois, United States of America; 5 Neuroscience Program, University of Illinois at Urbana-Champaign, Urbana, Illinois, United States of America; Nanjing University, CHINA

## Abstract

Many soft-bodied animals have extensive peripheral nervous systems (PNS) with significant sensory roles. One such, the sea slug *Pleurobranchaea californica*, uses PNS computations in its chemotactile oral veil (OV) in prey tracking, averaging olfactory stimuli across the OV to target likely source direction, or “stimulus place”. This suggests a peripheral subepithelial network (SeN) interconnecting sensory sites to compute the directional average. We pursued anatomy and connectivity of previously described ciliated putative sensory cells on OV papillae. Scanning electron microscopy (SEM) confirmed paddle-shaped cilia in clusters. Anti-tubulin and phalloidin staining showed connections to branching nervelets and muscle fibers for contraction and expansion of papillae. Ciliary cell processes could not be traced into nerves, consistent with sensory transmission to CNS via secondary afferents. Anti-tyrosine hydroxylase-stained ciliated cells in clusters and revealed an at least partially dopaminergic subepithelial network interconnecting clusters near and distant, connections consistent with PNS averaging of multiple stimulated loci. Other, unidentified, SeN neurotransmitters are likely. Confirming chemotactile functions, perfusible suction electrodes recorded ciliary spiking excited by both mechanical and appetitive chemical stimuli. Stimuli induced sensory nerve spiking like that encoding stimulus place. Sensory nerve spikes and cilia cluster spikes were not identifiable as generated by the same neurons. Ciliary clusters likely drive the sensory nerve spikes via SeN, mediating appetitive and stimulus place codes to CNS. These observations may facilitate future analyses of the PNS in odor discrimination and memory, and also suggest such SeNs as potential evolutionary precursors of CNS place-coding circuitry in the segmented, skeletonized protostomes and deuterostomes.

## Introduction

Many soft-bodied animals have an extensive peripheral nervous system (PNS) with significant roles in sensory integration. However, computations in the gastropod PNS can be surprisingly complex and are not yet well understood. For active foragers the peripheral sensory system is critically involved in chemotactile detection and discrimination.

In the basal nudipleuran *Pleurobranchaea californica*, chemotactile stimuli are dually processed: 1) to act at the feeding motor network to incentivize appetitive behavior, and 2) to directionally track stimulus source at the turn premotor network (Movie 1, Supplementary Information). Yafremava and colleagues [1,2], recording sensory nerves found that the PNS of the chemotactic oral veil (OV) averages multiple stimulated sensory loci to calculate a “place code” usable as a motor template for targeting turn responses. Those results suggested that the PNS could perform such computations through a layer of neuronal interconnections between the various sensor sites on the oral veil, perhaps through a lateral inhibitory network, which is a computational circuitry characteristic of sensory systems in arthropods and vertebrates, but hitherto unsuspected in the gastropod olfactory system. Moreover, previous work identified putative dopaminergic sensory cells in the sensory papillae of the OV and showed that appetitive effects of food stimuli were partially blocked by a dopaminergic receptor blocker [3]. We pursued the anatomy and physiology of the peripheral sensory network to further discover the nature of the sensory PNS.

The flow of chemotactile information at the oral veil begins with the sensory structures studied by Matera and Davis [4] with light microscopy and scanning and transmission electron microscopy. They located putative sensory cells with characteristic paddle-shaped cilia in clusters in known chemosensory areas of rhinophore, tentacle, and oral veil. We have revisited structure and function of the sensory PNS in the OV, beginning with the cilia clusters where the putative sensory cells are located [4,5]. We revealed the structures of the ciliary clusters and the well-organized nerve branches innervating them with scanning Electron Microscopy (SEM), immunostaining for tubulin and tyrosine hydroxylase, and phalloidin labeling for actin. A broad subepithelial meshwork of dopaminergic neural processes was found below the cilia clusters to run between clusters and receive branches of the sensory nerves. Stained axons of the ciliary clusters could not be traced beyond the meshwork into the nerve roots. We were able to record action potentials from the clusters and their excitation by both mechanical and appetitive chemical stimuli. However, spikes in sensory nerves did not correlate one-for-one with cilia spikes and likely represented secondary afferents.

These results are interpreted as evidence that a peripheral subepithelial network (SeN), at least partly dopaminergic, may receive primary chemotactile information and encode both stimulus source strength and direction to send to central networks mediating appetitive behavior and behavioral orienting turns.

## Methods

### Animals

Specimens of *Pleurobranchaea californica* (a hermaphrodite) trapped in the canyons of Monterey Bay, were purchased from Monterey Abalone Co. (Monterey, CA) and maintained in artificial seawater (ASW) at 11–12°C. 12 animals were used in these experiments. Animals were fed a few grams of squid flesh every 2–3 days, except that they were not fed for 3 days before use to bring their appetitive states into an intermediate state of satiation/hunger [6], in case that it might affect the sensory gain as well as central feeding network excitability [7]. All procedures involving animals were approved by the Institutional Animal Care and Use Committee of the University of Illinois at Urbana-Champaign.

### Scanning Electron Microscopy (SEM)

For tissue isolations, anesthetized animals were secured on a Sylgard-coated plate in chilled ASW. Papillae 2–3 mm in length and tentacle tips were excised from the OV and relaxed in MgCl_2_ (0.33 M, 10 mM HEPES, pH = 8.0) for three hours prior to fixation. Papillae were fixed in 2.5% glutaraldehyde in 0.1 M PBS at pH 7.6 for 4 hours at room temperature and washed for 2 hours in 2.5% sodium bicarbonate. A secondary fixation was for 3 hours at room temperature in 2% osmium tetroxide in 1.25% sodium bicarbonate. Tissues were next rinsed 3X in distilled water and dehydrated in ethanol. For critical point drying we used the Samdri-790 (Tousimis Research Corporation). After drying, tissues were processed for metal coating on Sputter Coater (SPI Sputter) using the gold/palladium target. SEM analyses and photographs were done on a NeoScope JCM-5000 microscope (JEOL Ltd., Tokyo, Japan). In all, seven groups of oral veil tissues were processed independently through fixation, drying, metal coating and imaging, each containing between 6 and 10 papillae-containing pieces dissected from the oral veils of two healthy adult *Pleurobranchaea*.

### Immunocytochemistry

Tissues from 3 animals were fixed in 4% paraformaldehyde in 0.1 M PBS overnight at 4°C, then rinsed 3X in PBS, 30 minutes each time. For whole-mounts, tissues were treated with type XIV protease for 30 mins to improve antibody penetration. Other tissues were embedded in low-melting point agarose in 0.1X PBS and 1 mm thick sections were made on vibratome. Fixed and rinsed tissues were preincubated overnight in a blocking solution (6% goat serum in PBS) and subsequently incubated in primary antibodies at 1:40 dilution for 48 hours at 4°C (rat monoclonal antibody, AbD Serotec Cat# MCA77G, RRID: AB_325003, against alpha subunit of tubulin). Following a 6 hour total PBS wash, the tissues were incubated for 12 hours in secondary antibodies–goat anti-rat IgG antibodies (Alexa Fluor 488 conjugated; Molecular Probes, Invitrogen, Cat# A11006, RRID: AB_141373) at a final dilution of 1:20.

To detect catecholaminergic neural elements, a mouse monoclonal antibody against tyrosine hydroxylase was used (Immunostar; Cat# 22941, RRID: AB_572268). Primary antibody dilution was 1:60 and incubation time was 48 hours at 4°C. Following primary antibody incubation, tissue was washed in PBS and incubated in secondary antibodies–goat anti-mouse IgG (Alexa 488 conjugated; Molecular Probes; Cat# A-11001, RRID: AB_2534069) at a final dilution 1:30.

Muscle fibers were labeled with phalloidin (Alexa Fluor 568 from Molecular Probes), which binds to F-actin. After washing in PBS following the secondary antibody treatment, tissues were incubated in PBS with phalloidin at a final dilution 1:80 for 4 to 8 hours, and then washed in several PBS rinses for 6 hours. To stain nuclei, the tissues were mounted in VECTASHIELD Mounting Medium with DAPI (Cat# H1200). The preparations were viewed and photographed using Nikon C1 Laser Scanning confocal microscope.

Rat monoclonal antityrosinated alpha-tubulin antibody used in this study was raised against yeast tubulin and has been successfully used before on species of ctenophores and hydrozoans to label neural elements [8,9]. The specificity of immunostaining was tested by omitting either primary or secondary antibody, for which cases no labeling was detected. The mouse monoclonal antibody against tyrosine hydroxylase (TH antibody) was previously used in *Pleurobranchaea* [3]. We successfully repeated the same tests on the specificity of immunostaining by omitting either the primary or the secondary antibody. There was no significant autofluorescence. Here we use the term TH-li (TH-like immunoreactivity). For immunostaining with the tubulin antibody, there were 16 independent groups of oral veil tissue sections processed in the protocol and confocally imaged. Each group contained between 10 and 20 sections from 4 animals. The work was performed in two separate Summer seasons with similar results. For immunostaining with TH antibody, there were 11 independent runs through the staining protocol, each containing between 10 and 20 veil sections from 3 animals done over two Summer seasons, with similar results.

### Electrophysiology

Prior to recordings of papillae in intact animals, they were anesthetized by injection of one-third body volumes of 0.33 M MgCl_2_ and 10 mM HEPES, pH = 7.6. Excitability of sensory units in the high internal Mg2+ was apparently maintained by the normal ASW in the recording suction electrodes. At 20–30 minutes, relaxed animals were pinned on a Sylgard coated plate in ASW at 11°C. Signals from papillae were recorded extracellularly with suction electrodes, differentially AC amplified and registered by a data acquisition system (PowerLab 8/30, AD Instruments, Dunedin, New Zealand). LabChart 7.3 (ADInstruments) was used to store and analyze the data. Signals were sampled at 20kHz with a 10Hz high-pass filter and a 1kHz low-pass filter. Where sensory nerves were also recorded with papillae, the oral veil was isolated from animals cold-anesthetized at 4°C and pinned in ASW with sensory nerve open to suction electrodes. In other experiments the CNS remained attached to the oral veil while the LOVN sensory nerve was recorded *en passant* simultaneously with recordings of papillae.

**Fig 1:**
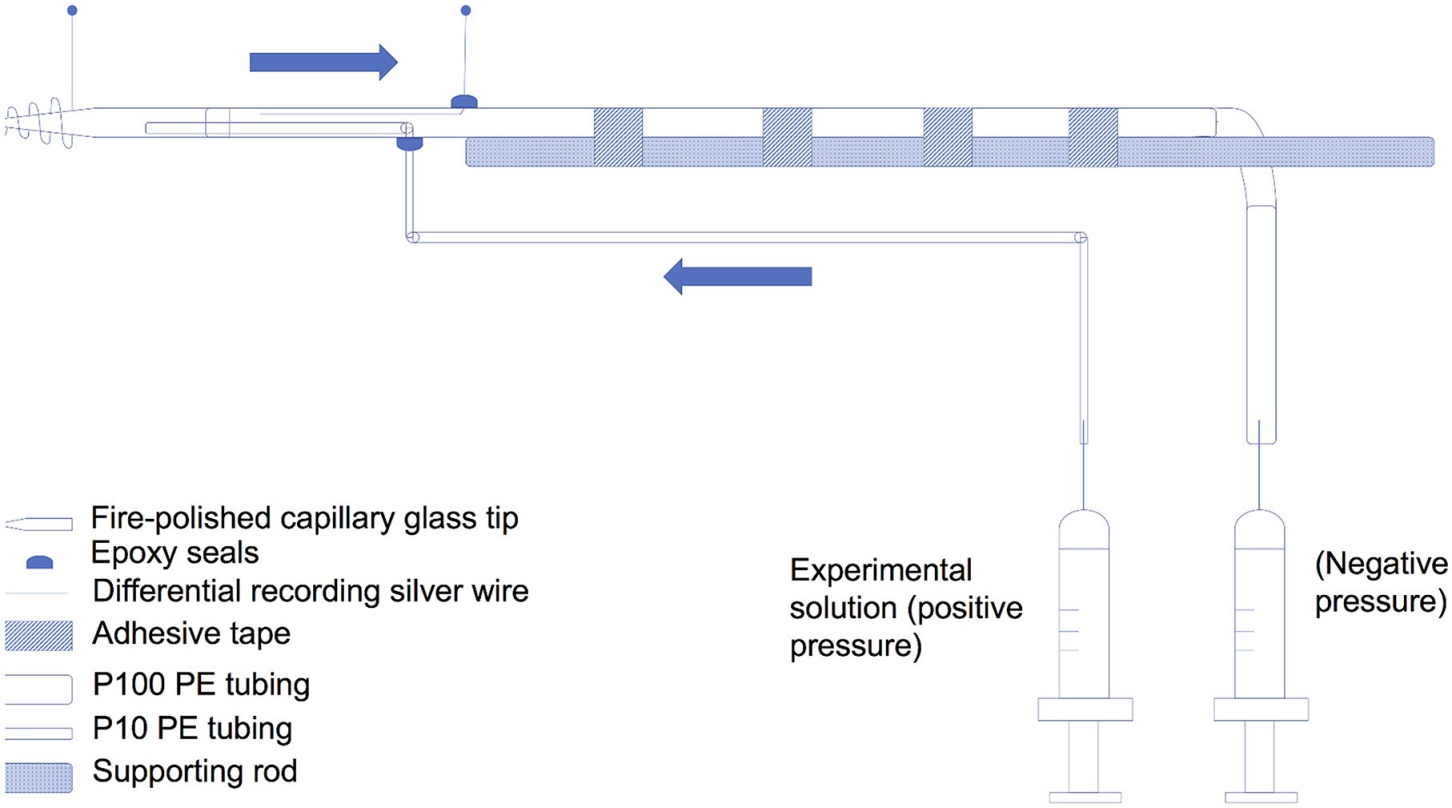
The perfusion suction electrode allowed local perfusion of chemicals at papillae while maintaining a stable recording. During recordings solutions could be perfused from the PE 10 tubing into the main body of the electrode while negative pressure in the electrode (and a high shunt resistance) was maintained.

A special suction electrode was devised (Yichen Liu) to allow perfusion of chemicals while recording the oral veil papillae (Fig 1). The body of the electrode was PE100 polyethylene tubing with an inner recording silver wire; a second differential electrode wire coiled around the tip from outside. Perfusion saline was cooled to the same temperature as the bath and entered the suction electrode through PE10 tubing within the electrode’s main body. Fire-polished glass capillaries of different diameters were attached to the P100 tubing tip to record differently sized papillae. When the electrode was placed on the multilobed papilla, gentle suction was applied to increase shunt resistance at the tip for recording. To test the mechano- and chemosensitivities of the papilla, the perfusion electrode was washed sequentially with ASW and 10 or 100 mM betaine (trimethylglycine HCl; Sigma-Aldrich) in ASW with 10 mM HEPES at pH 8.0. A transient mechanoceptive “on” response caused by perfusion initiation was fully adapted at 6–10 seconds later when the betaine stimulus arrived at the papilla (see Results). Blue food dye added to the betaine solution served as an indicator for when the betaine solution reached the papilla. Neither intact animals nor papillae were responsive to the blue dye in ASW alone.

### Data analysis

Recordings were analyzed by the spike analysis modules in LabChart 7. Background noise levels were smaller than 4 μV, and spikes were registered with amplitudes larger than 8 μV. Activities recorded 3 or 5 seconds before treatments were used as baseline and all experimental responses were normalized to the baseline. Wilcoxon Signed Rank tests tested significances of treatment effects. Statistical analyses were carried out in Python 3.10.

## Results

**Fig 2:**
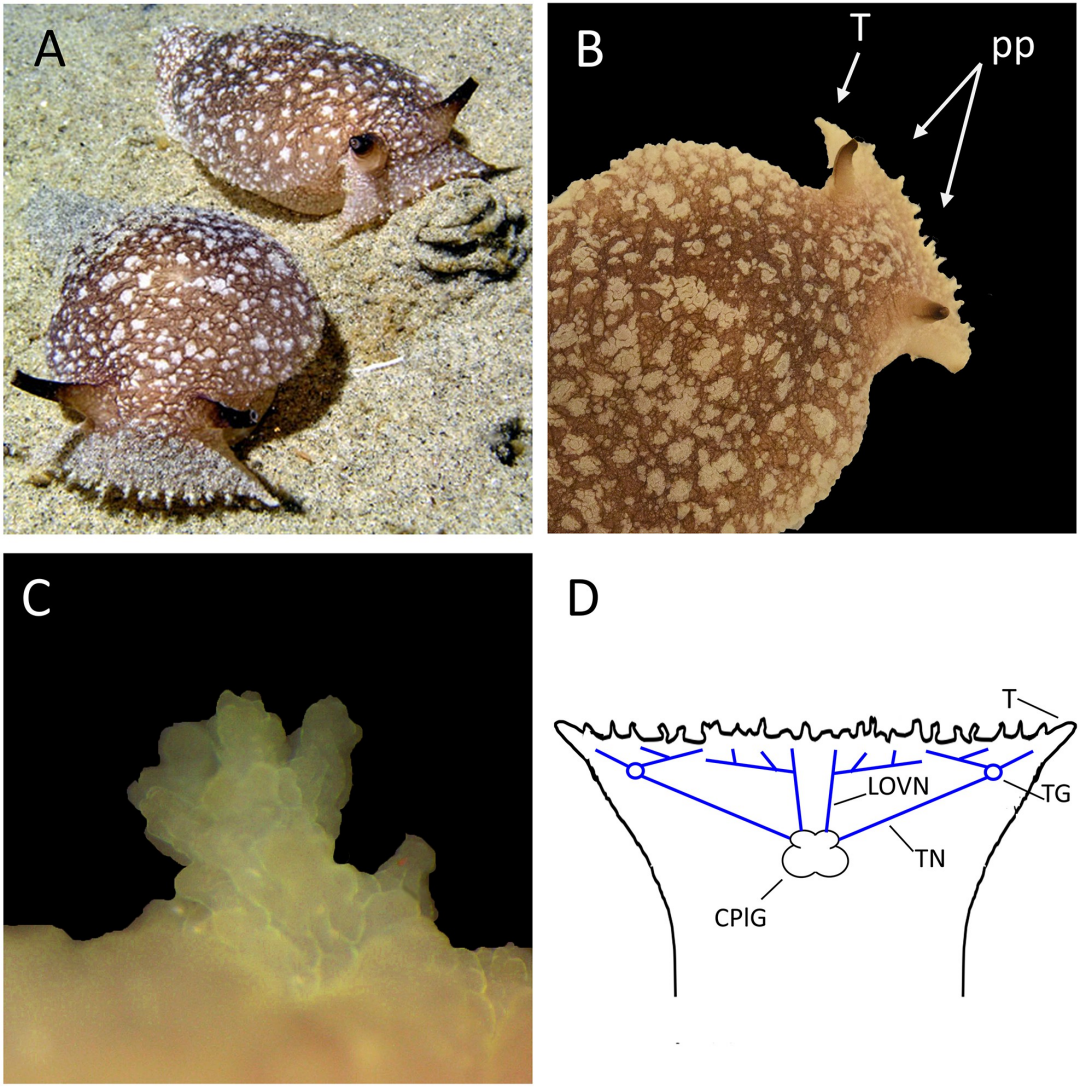
The oral veil (OV) with papillae, tentacle, and their innervation. **A**: *Pleurobranchaea* foraging (La Jolla Shores, CA.; photo by Tracy Clark). B: Papillae (pp) on the oral veil. T, the tentacle portion of the oral veil **C**: A single papilla. Papillae are multi-lobed and vary in size, with larger papillae near the midline of the oral veil. **D**: Branching of the two sensory nerves, the large oral veil nerve (LOVN) and the tentacle nerve (TN) innervating both the tentacle (T) and adjacent oral veil. The nerves overlap in their innervation of the OV and bring sensory information to the cerebropleural ganglion (CPlG).

Sensory papillae of the oral veil/tentacle complex of *Pleurobranchaea* are shown in Fig 2. Papillae are under muscular control and vary in states of extension and contraction during exploration, aversive behaviors, and quiescence.

### Scanning electron microscopy

**Fig 3:**
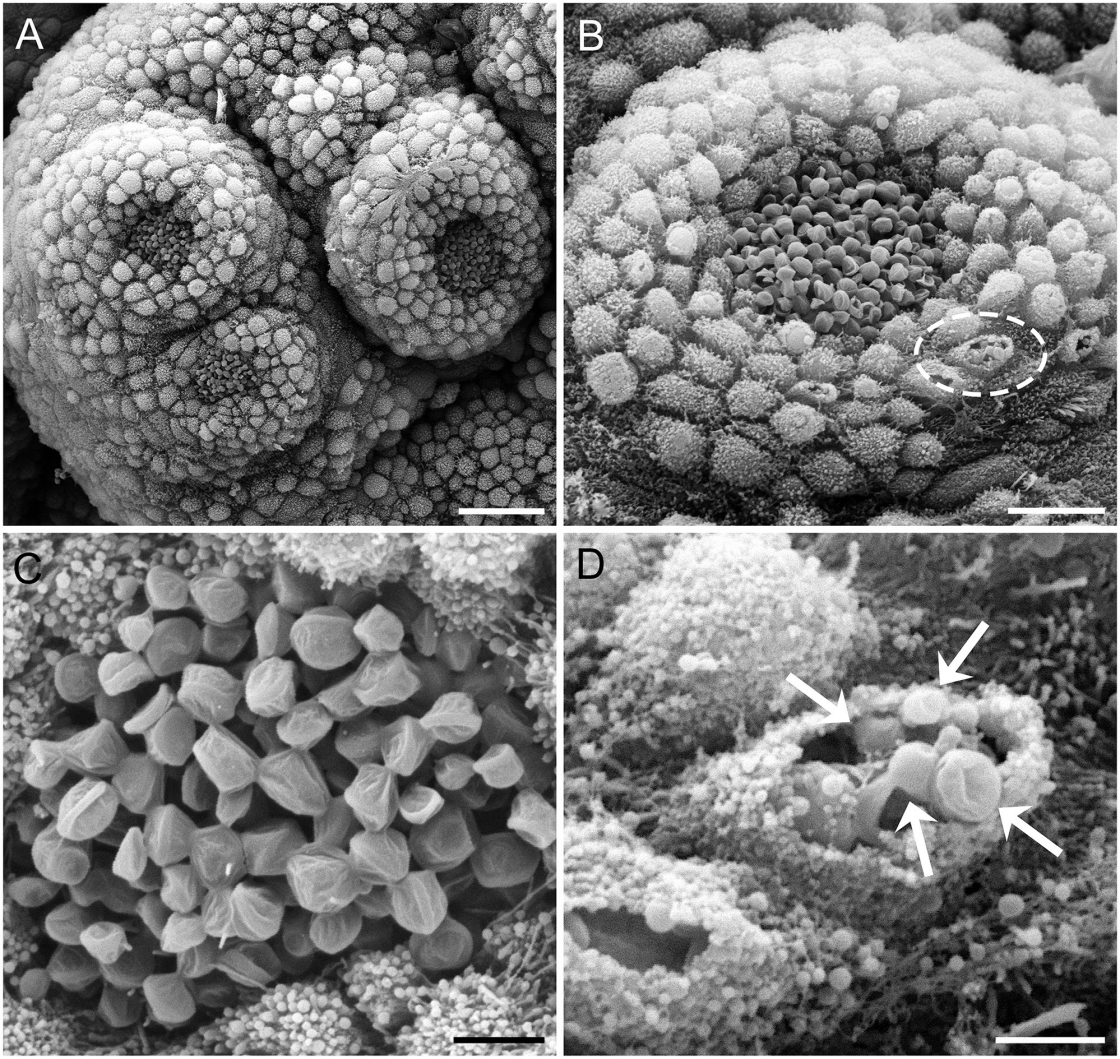
SEM images of papillae surfaces. **A**: Three clusters of cilia on a lobe. **B**: Each cluster is surrounded by numerous tubercles, some of which in this image are ruptured (dashed ellipse). **C**: There are about 100 cilia in each cluster, with all showing the dilated heads of paddle cilia. **D**: Paddle cilia-like structures (arrows) are observed within the several broken tubercles of B, above. Scale bars: A, 25 μm; B, 10 μm; C, 3 μm; and D, 2 μm.

Despite 3 hours of relaxation in high Mg^2+^, some muscle contraction occurred during tissue fixation. Most cilia imaged were on partially retracted lobes, but their tips were clearly revealed. A single papilla could have from 8 to 12 lobes, where each, depending on its size, would have between 10 and 20 clusters of cilia (part of a lobe with three cilia clusters is seen in Fig 3A). Individual clusters consisted of up to 100 cilia (Fig 3B and 3C). Most cilia showed characteristic discoid dilations at the tips with ridged structure (Fig 3C), corresponding well to descriptions of Matera and Davis [4].

**Fig 4:**
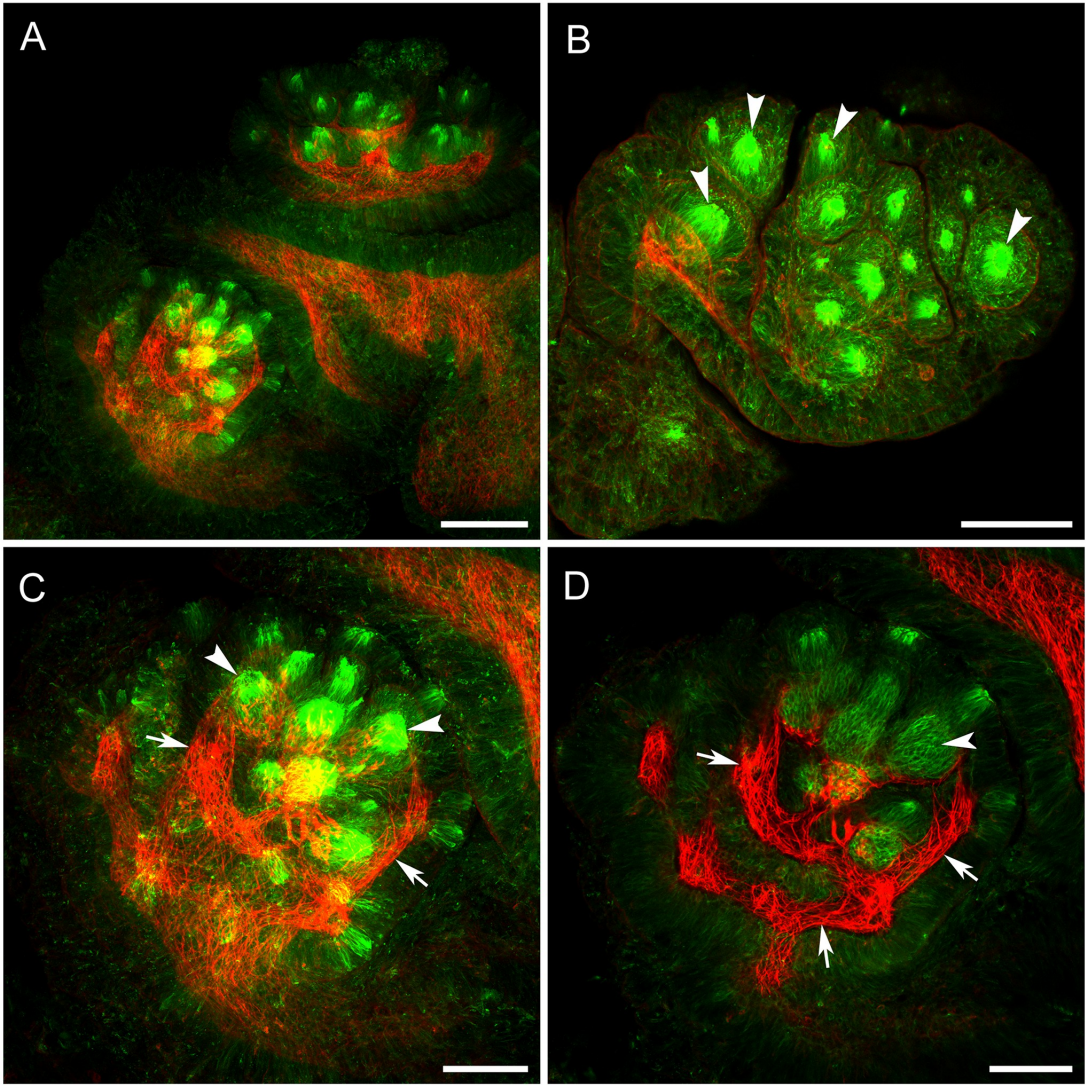
Anti-tubulin and phalloidin staining of papillae in whole-mounted tissues. Cilia clusters on the lobules are green (anti-tubulin). Muscle is red (phalloidin). **A-C**: Antibody and phalloidin penetrated only the superficial level of the tissues, probably through the clusters, revealing the clusters of sensory cilia in the lobules (arrowheads) and the adjacent muscle fibers (arrows). Finer cilia surround the central bundles. Muscle fibers lie immediately beneath the cilia clusters. **D**: A confocal section of C showing off the glomerular tangles below the cilia (arrowhead) and muscle fibers (arrows). Scale bars A, 200 μm; B-D, 50 μm.

Many small, round tubercles (~ 1 μm dia.) in the skin surrounded the central cilia clusters. A few of the tubercles were damaged, with holes in their surfaces revealing cavities in which apparent cilia could be seen like those in the central clusters, except smaller (Fig 3D). These were distributed to possibly correspond to the scattered fine threads of anti-tubulin staining in the epithelium around the clusters of cilia in the immunostained whole-mount tissues (Fig 4B).

### Immunohistochemistry

The skin of whole-mounted tissues resisted antibody penetration even after protease digestion. However, areas close to the surface were labeled by tubulin AB and revealed multiple clusters of cilia in the papilla (Fig 4). Those multiple clusters of cilia presumably represented sensory afferents and were grouped together in lobules corresponding to the multi-lobed papillar morphology. Phalloidin, being a much smaller molecule, penetrated the tissue easily and stained the muscle fibers immediately beneath the cilia clusters, spiraling towards the base of the papilla (Fig 4). These muscles are likely responsible for the protraction and retraction of lobes and entire papillae in response to different stimuli. The muscle fibers from the cilia clusters formed an expanded capsule and interconnected clusters.

**Fig 5:**
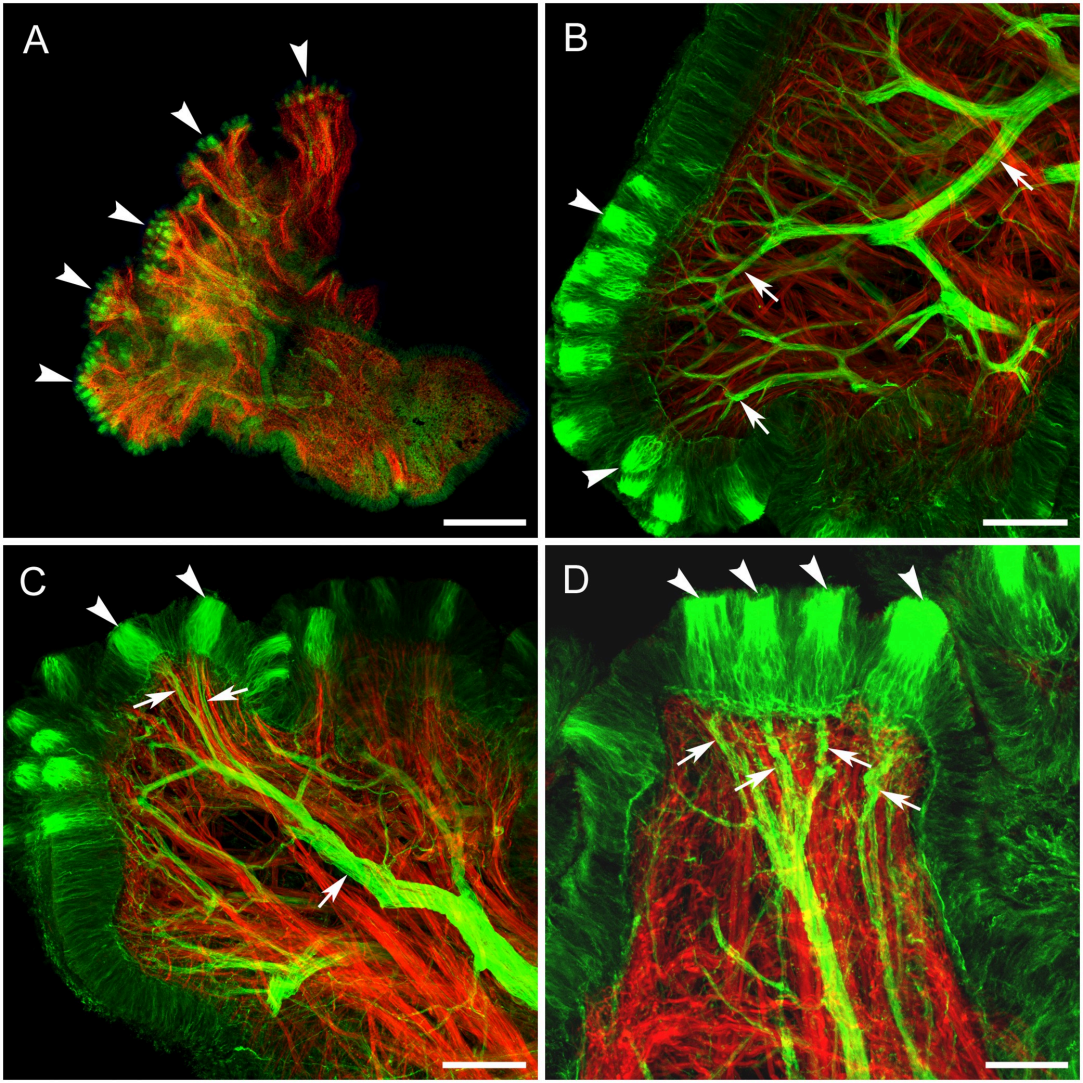
Sections through oral veil papillae and tentacle stained with anti-tubulin AB (green) and phalloidin (red), showing nerve and muscle structures, respectively. **A**: Horizontal section of a papilla at relatively low power. Arrowheads indicate papillar lobules with cilia clusters. **B, C**: Extensively branching nerves (arrows) approach clusters of cilia (arrowheads, which are also labeled by anti-tubulin AB. **D**: Higher magnification shows that each cilia cluster (arrowheads) is innervated by a small nerve branch (arrows). Scale bars: A, 500 μm; B, C: 100 μm; D, 50 μm.

By cutting through the center of the papilla and labeling the thin sections, the problem of antibody penetration was completely removed. In sections, tubulin AB labeled extensively branching thick nerves with their fine endings reaching the skin and innervating clusters of cilia (Fig 5). The cilia clusters of the papillae, separated from each other on the lobules, contrasted with the dense fields of clusters on tentacle. Each cluster of cilia had a thin nerve branch connected to it (Fig 5C and 5D). Bundles of phalloidin-stained muscle fibers ran from the walls of the papilla lobes, where the cilia clusters were located, to the base of each papilla and were likely responsible for retraction of papillae responding to different stimuli.

### Tyrosine hydroxylase immunostaining

We expanded on the detail of TH-li in the oral veil periphery originally shown by Brown et al., [3]. TH-li can mark neurons either dopaminergic or norepinephrinergic. As norepinephrine is rare in protostomes (but see [10]), where arousal functions appear to be carried out by octopamine, and the D2 receptor blocker sulpiride suppresses appetitive chemotactile stimulation in *Pleurobranchaea*’s oral veil [3], we presume that TH-li here is marking dopamine containing neurons.

**Fig 6:**
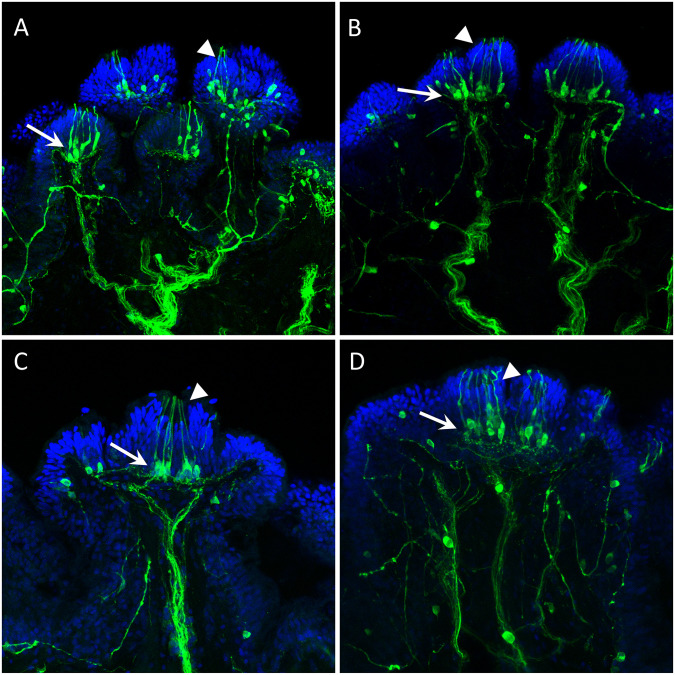
TH-li in branching nerves and sensory clusters. TH-li staining of cilia clusters and nerves is green; DAPI staining of cell nuclei in the epithelium is blue. **A, B**: Thick branching TH-li nerves (arrows) approach the clusters of cilia (arrowheads) described earlier. Note individual TH-li neuronal cell bodies spread around nerve branches and lateral branchlets near cilia clusters’ bases. **C, D**: Several TH-li cell bodies (arrows) each produces a single cilia-like projection to the surface. These presumed receptor cells lie at the base of the cilia cluster (arrowhead). Neuropil-like areas are visible below clusters. An axon of a cell body of these TH-li cells at the left arrow in C branches laterally. Scale bars: A and B, 100 μm; C and D, 50 μm.

Fig 6 shows TH-li innervation of the cilia clusters and the distributed dopaminergic neurons and processes in the PNS. Extensively branching thick nerves approach near the clusters of cilia on the surface. These nerves are TH immunoreactive (Fig 6A and 6B), although it is not known what fraction of the nerve axons are immunoreactive. There are also individual TH-li neuronal cell bodies scattered around the central part of the papillae between nerve branches. Those neurons do not obviously innervate the cilia clusters themselves, but their processes joined the axon bundles with apparent trajectories toward the CNS in the sensory nerves. Further, at the base of each cluster of cilia described earlier, there is a group of 10 to 15 TH-li cell bodies, containing 10 to 15 neuronal cell bodies of sizes about 10 μm. Each of these TH-li cell bodies has a single cilium projection running to the surface and embedded inside each cilia cluster (Fig 6A, 6B and 6D). On the proximal side of their cell bodies the axons of the TH-li cells are observed to join the subepithelial plexus of TH-li processes. However, their axons could not be traced beyond the subepithelial network into the afferent nerves.

**Fig 7:**
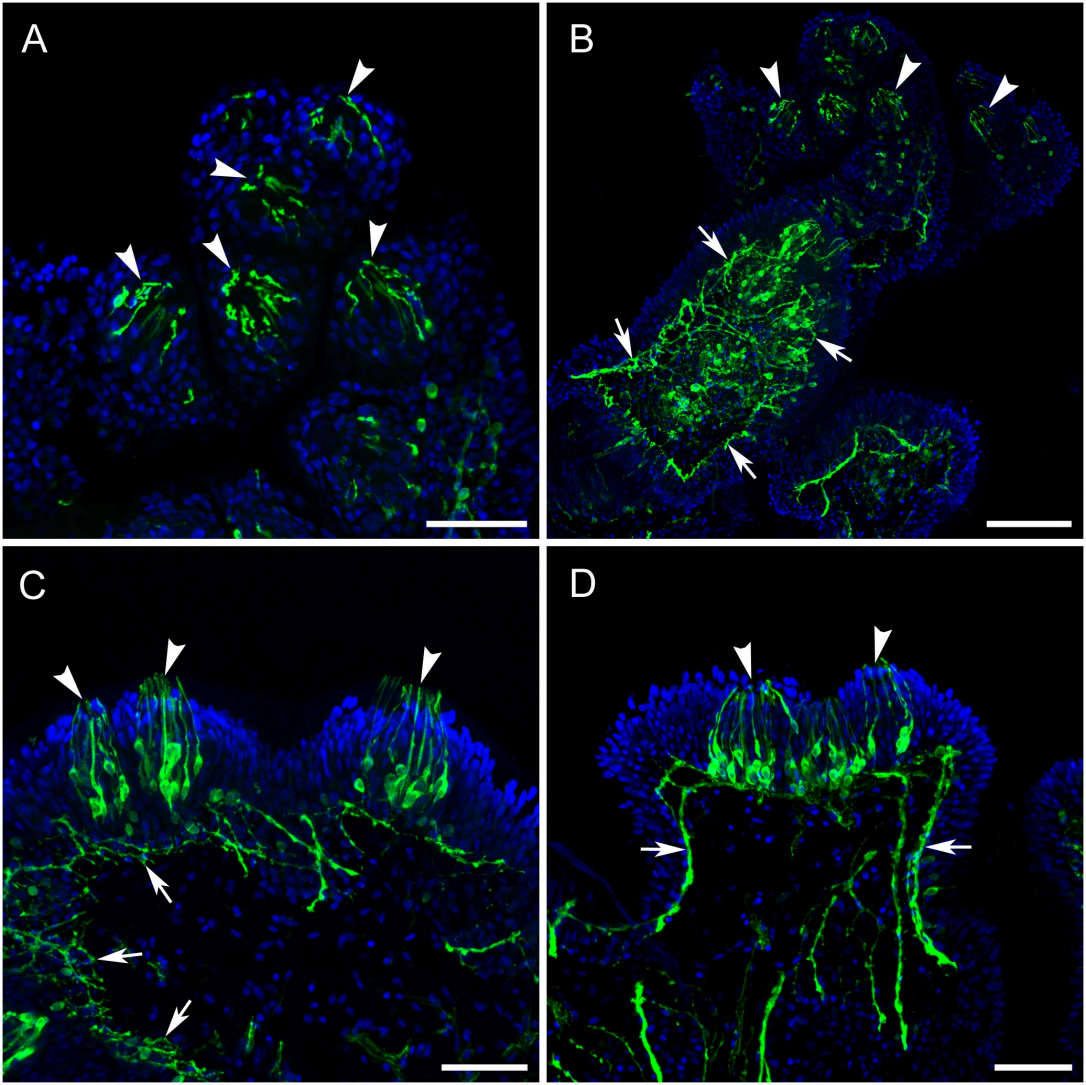
The SeN spreads under the skin of the papillae between neighboring cilia clusters. **A**: A horizontal section cut through the skin shows the TH-li cilia clusters (arrowheads). **B**: A horizontal section cut a little deeper reveals the subepithelial layer with a distinct and very dense network of TH-li neurites and neuronal cell bodies (arrows). **C**: A cross section of the papillae. The subepithelial TH-li neuronal network (arrows) spreads under the skin of the papillae, interconnecting the neighboring clusters of cilia (arrowheads). **D**: Nervelets (arrows) coming from cilia clusters (arrowheads) with lateral paths toward other clusters. Scale bars: A, C, and D, 50 μm; B, 100μm.

When sections were cut close to the papillae surface and across the papilla body wall, a TH-li element was revealed as a SeN of neuronal cell bodies and neurites. Just under the skin was a thin-layered dense meshwork of TH-li neurons and fine neurites, which spread around the entire papillae, presumably connecting the neighboring clusters of cilia and other surface sensory elements (Fig 7).

### Electrophysiology

Problems with mucus clogging the electrodes and loss of recording by spontaneous movements of peripheral muscle were rare. Reliable suction electrode recordings from the ciliary clusters of the papillar lobules on *Pleurobranchaea*’s oral veil were obtained consistently. Recordings from papillae showed spontaneous spikes of various sizes and typically rather long in duration (durations at half-amplitudes of 10–30 ms), and smaller in amplitude compared to units recorded in the nerves with similar electrodes. However, spike amplitudes recorded with suction electrodes from different structures and times may not be comparable as shunt resistances can differ.

**Fig 8:**
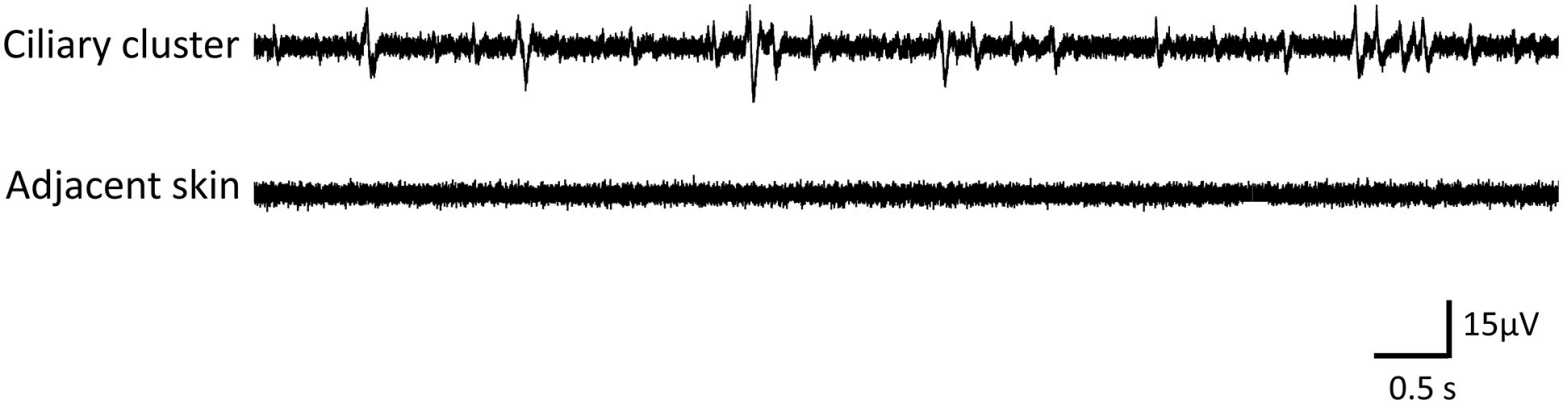
Spontaneous spiking activity in a focally recorded cilia cluster and the lack thereof in adjacent skin.

To address a concern that papillar recordings were monitoring muscle discharges instead of sensory cilia clusters, we compared them against those of adjacent skin (Fig 8). While long-duration and small-amplitude spontaneous spikes were recorded from the clusters, almost no discernible units were present in adjacent skin recordings. Units from papillae spontaneously fired at an average of 3.64 ± 0.54 Hz (N = 27 papillae measured in 3 animals), contrasting with spontaneous activity averaging only 0.15±0.05 Hz (N = 19 measures in 3 animals) of activities were detected in the skin. The origin of occasional activity in skin recordings is unknown but could be in solitary chemosensory cells like those sensitive to the aversive chemical taurine [11]. Recorded action potentials from the clusters had at least three possible sources: the sensory cilia, the muscle fibers associated with the clusters, and the SeN at the base of the clusters. While it remains to be finally determined, we expect that most spikes came from the ciliated cells that resemble the characteristically excitable mammalian olfactory cells [12], since subepidermal cells of the plexus or muscle fibers would have been likely less excitable in Mg2+ anesthetized animals, while excitability was maintained from the cilia by the normal external saline.

**Fig 9:**
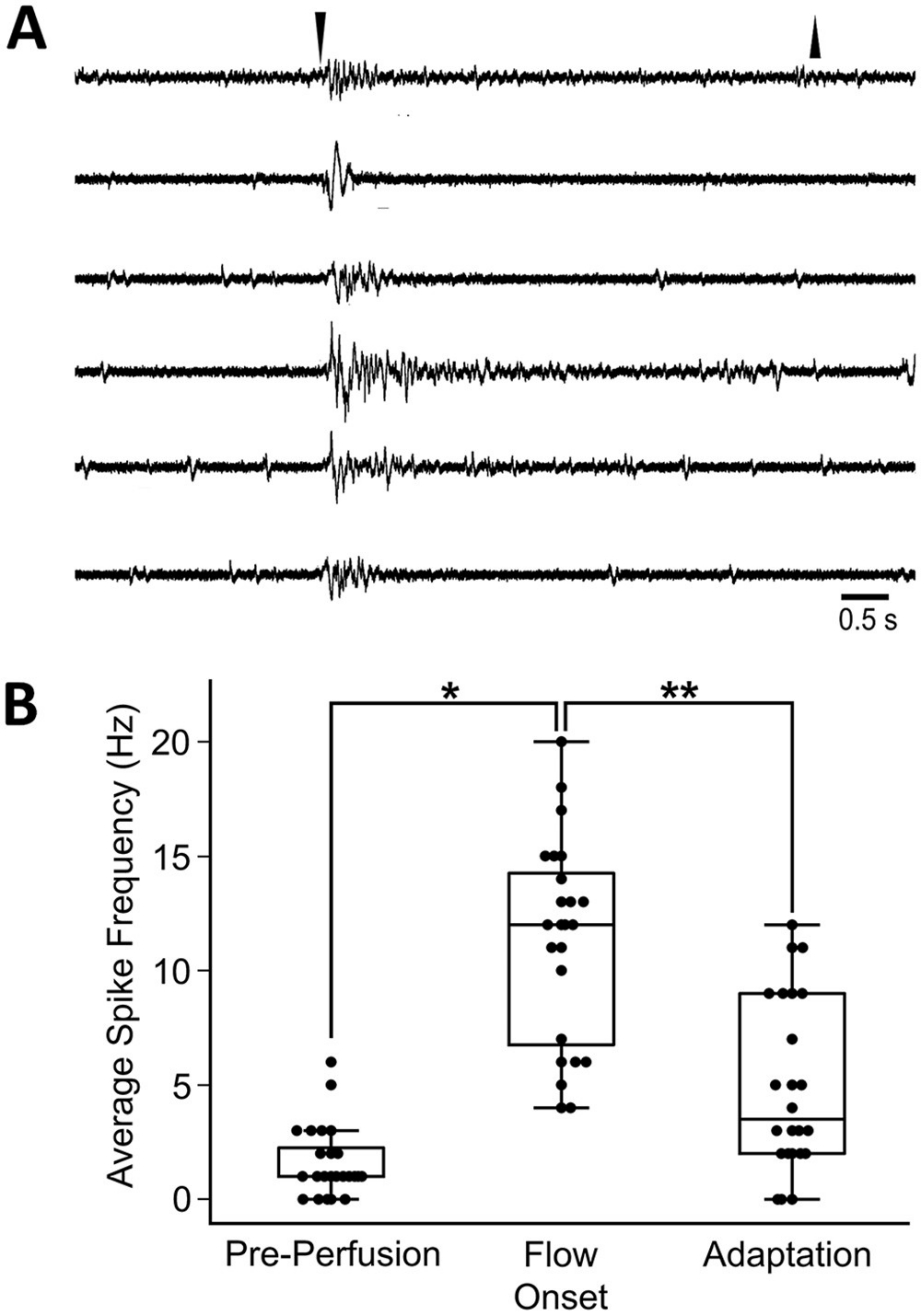
Mechanoceptive responses of papillae at perfusion onset. **A**: “On” responses from papillae at onset of ASW flow. The records are from 24 recordings of different papillae in three different animals. Immediately upon the step change in pressure in the suction electrode with ASW perfusion, brief bursts of spikes occurred. Responses quickly adapted while flow was maintained. Recordings from skin adjacent to papillae showed little spontaneous and no induced mechanosensitive activity (not shown). Perfusion offset (downward arrowhead) was 5 seconds after onset (upward arrowhead) for most (19 out of 24) recordings, and for 5 recordings it was 2 seconds after onset. **B**: Papillar spike frequencies for 1 second time-windows before and after onset of perfusion, and before perfusion offset (box plots show distributions of the firing frequencies, including mean, interquartile distribution, and outliers). Averaged pre-perfusion baseline firing frequency was 1.625 ± 0.31 Hz (median: 1.0 Hz, standard error: 0.311), increasing to 11.30 ± 0.90 Hz (median: 12.0 Hz, standard error: 0.897) at perfusion onset. Spike activity rapidly adapted to lower firing frequency (4.92 ± 0.75 Hz; median: 3.5 Hz, standard error: 0.752) measured at 1 second before perfusion offset. These effects were significant (Wilcoxon Signed-Rank tests p < 0.00002 for perfusion onset response vs. pre-perfusion (*) and for perfusion onset vs. adaptation (**), and slightly less significant p < 0.0003 for pre-perfusion vs. adaptation).

Papillae were mechanically responsive to water flow. Transient “on” bursts of spike activity occurred to onset of ASW flow and rapidly declined to pre-flow levels (Fig 9). On responses typically lasted 1–3 s where the average firing frequencies increased more than 3X from baseline and units appeared that were appreciably larger than spontaneous units in the pre-stimulus period. Spiking activity rapidly adapted to lower firing frequency during the perfusion. “Off” responses were not observed. Recordings of adjacent skin under the same circumstances yielded no spiking responses (not shown).

**Fig 10:**
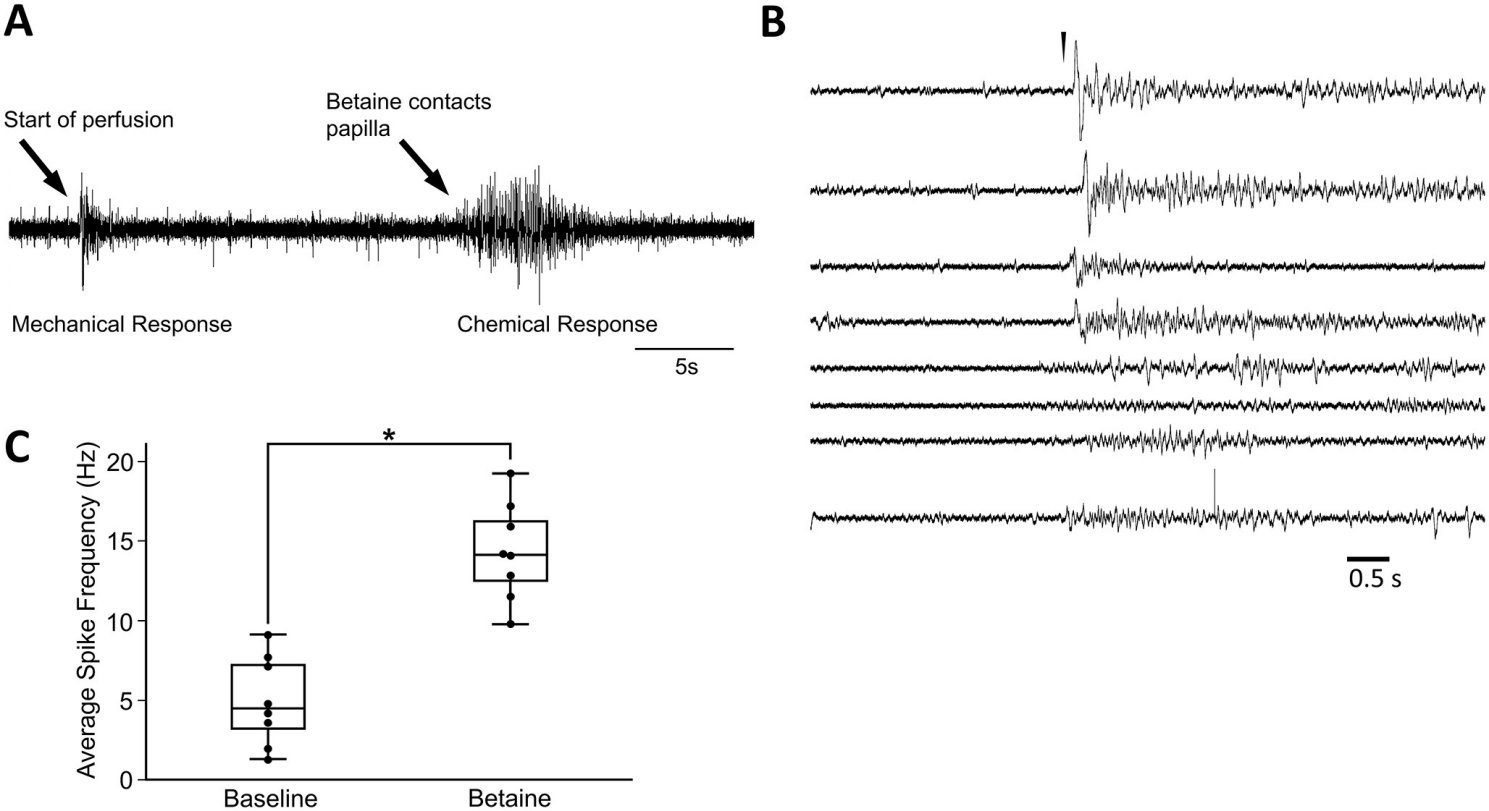
Chemoceptive responses of papillae to betaine perfusion (0.1M trimethylglycine). **A**. The record shows the latency from activity induced by perfusion onset to activity induced by betaine stimulation. **B**. Betaine stimulation (arrowhead) induces spike activity. Shown are 8 recordings of different papillae from 3 different animals. The arrowhead indicates betaine contact with papillae. **C**. In recordings of B, comparing activity 3 seconds before and after papillar contact, betaine increased spike frequency from an average baseline of 4.04 ± 0.68 (median: 4.00 Hz, standard error: 0.678) to 14.04 ± 1.1 Hz (median: 13.33 Hz, standard error: 1.01) with significant difference (* p < 0.008, Wilcoxon Signed-Rank test).

Fig 10A shows a latency between mechanosensitive and chemoceptive responses at the beginning of perfusion and arrival of appetitive stimulus at the recorded papilla. Papillar chemoceptive sensitivity was first noted in pilot observations where spike activity was markedly increased by adding drops of crab blood (*Pachygrapsus sp*.) to a dish in which papillae from an excised oral veil were being recorded. The comparisons were inexact because the suction electrodes had to be temporarily removed to allow the blood infused saline to reach the recorded areas. Thus, the perfusion electrode was made to allow continuous recording during chemical stimuli. Chemosensitivity was tested by perfusing the appetitive chemical betaine (trimethylglycine) at 0.1 M (Fig 10A–10C). The betaine solution was perfused relatively slowly for sufficient temporal separation between the mechanical and the chemical responses. Betaine contacted papillae 6–20 seconds following the initial “on” excitation at perfusion onset, at which times the mechanical responses were fully adapted. Spikes occurred almost immediately as the solution contacted papillae, as indicated by the added blue food dye. Control recordings from adjacent skin yielded no responses (not shown). Spike frequency adaptation to the betaine stimulus was apparent over 5–10 seconds.

**Fig 11:**
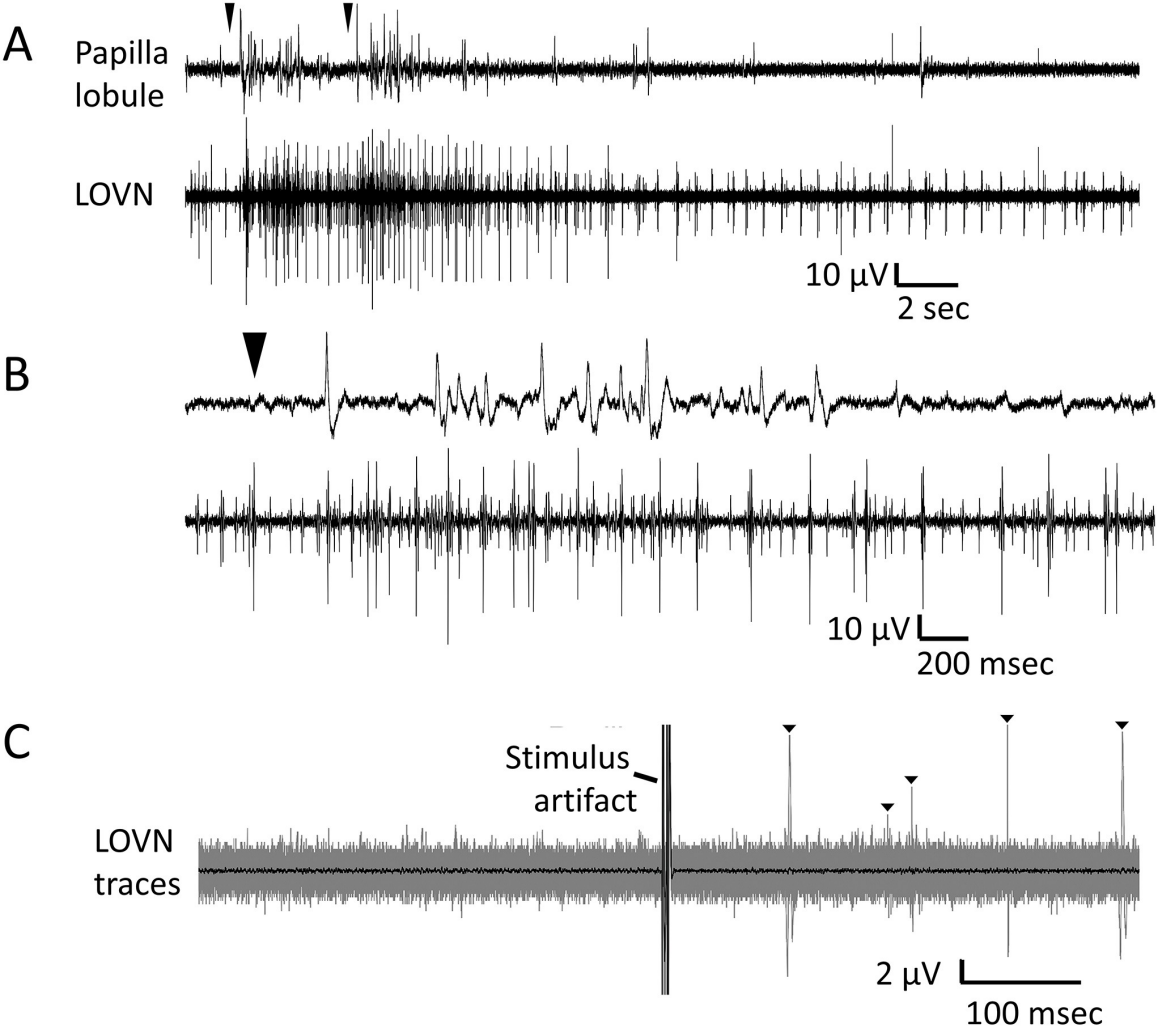
LOVN afferent spikes originate in the subepithelial network. Orthodromic action potentials originating in papillae were not identifiable in the sensory LOVN. **A**. A gentle water jet from a Pasteur pipet was applied twice (arrows) at the midline of the isolated oral veil, likely stimulating multiple papillae mechanically. Recordings of a single papilla (upper trace) and the LOVN (lower trace) show typical loosely correlated activities. **B**. An expanded record of A, showing difficulty in identifying common spikes. Recording sites were separated by ~2 cm of nerve. The record also shows the relatively longer duration spikes of the ciliary clusters relative to nerve. Other parts of the expanded records are available in Supplementary Information. **C**. Stimulating a single papilla with a suction electrode (0.2 V, 4 msec duration, single stimuli at intervals of 4–10 sec) over 55 trials failed to drive correlated spikes in the LOVN with similar latencies but drove a few larger spikes at long latencies (triangles). *Larger trace*: Overlays of the 55 trials. Spiking post-stimulus was significant (Wilcoxon Signed Rank test, using right-tailed Z distribution, p = 0.018). Latencies of these large-amplitude spikes were quite variable, as expected if synaptically activated. *Smaller dark trace*: The average of the 55 trials in which, if present, smaller, orthodromically driven spikes should have additively emerged from the noise but did not.

Thus, LOVN spikes are likely to originate in the SeN, driven synaptically by papillar input. Units recorded from the cilia clusters could not be identified in recordings of the sensory nerves in isolated oral veil preparations (Fig 11A and 11B). The TN and LOVN nerves have relatively few recordable axons; judged by spike sizes perhaps 12–18 recordable afferent axons in each [2,3], none of which could be related one-for-one to spikes observed in the papillae. Stimulating papillae with suction electrodes elicited a few action potentials recorded in the LOVN at variable and relatively long latencies (Fig 11C) atypical of directly transmitted spikes but consistent with one or more intervening synapses.

**Fig 12:**
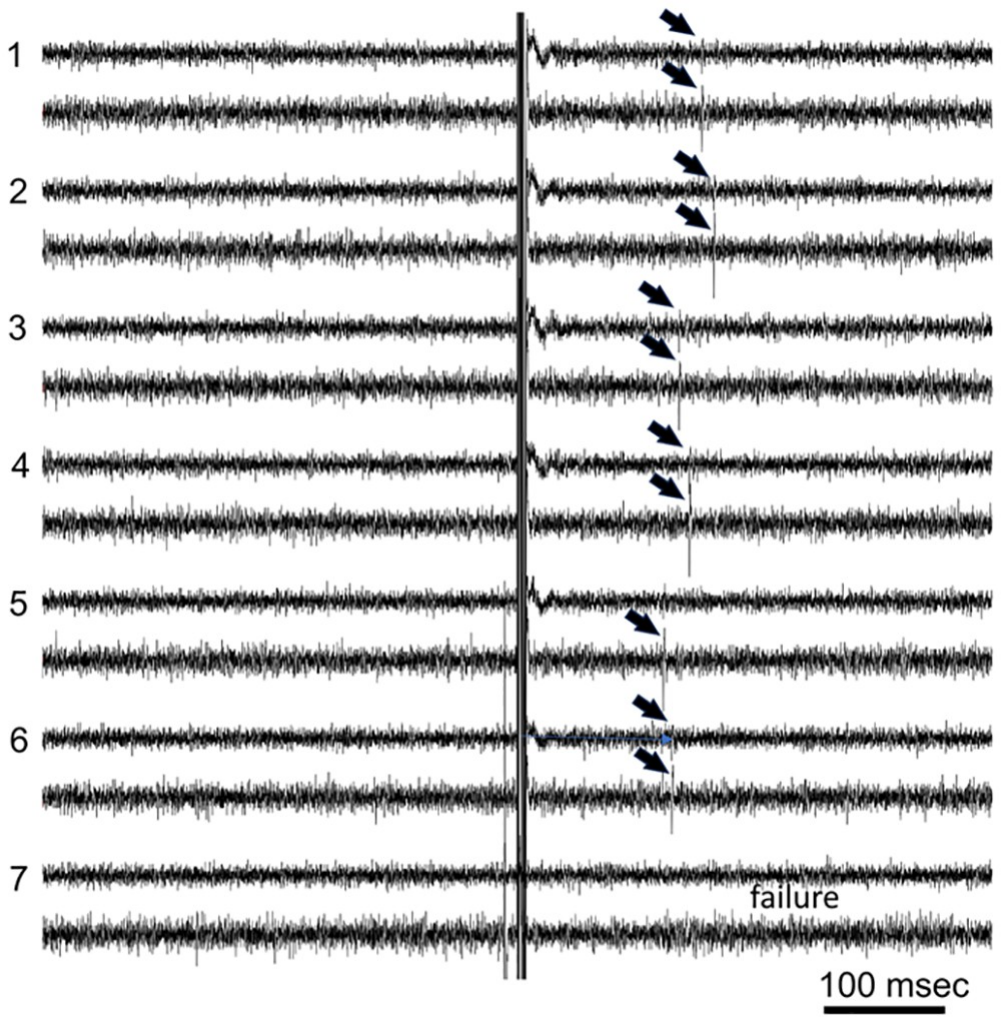
Efferent spikes in papillae from LOVN stimulation. Shown are 7 of 30 records where two papillae were recorded simultaneously during single LOVN stimuli. Following the stimulus artifacts in mid-traces, single spikes (arrows) appeared in papillae at variable latencies. The spikes in the papillar pair had nearly identical latencies. Spikes sometimes failed in both pairs (record 7), or more often, either failed to appear in the papilla of the upper trace pairs (e.g., record 5) or failed to fully invade (record 6).

Electrical stimulation of the LOVN while recording papillae in three experiments did not cause spiking in papillae or caused single, common spikes between papillae (Fig 12). In the single experiment where LOVN induced papillar spikes, the spike latencies showed variability consistent with either synaptic mediation or spike blockade at diverging axonal branch points [13]. These observations are consistent with absence of afferent axons in the sensory nerve from the papillar ciliary clusters, and further that the stimulated spikes recorded simultaneously in different papillae may normally originate centrally and may perhaps neuromodulate SeN and sensory cells.

## Discussion

These results add usefully to the anatomy and physiology of *Pleurobranchaea*’s oral veil sensory apparatus, and they test the sensory functions of the papillae originally inferred by Matera and Davis [4]. Moreover, they are consistent with a SeN underlying the oral veil’s ability to average chemotactile stimuli across the sensory papillae to compute the likely direction of the stimulus source [2]. We were able to confirm that the likely primary sensory cells have cilia with expanded paddle-shaped tips in the clusters on lobules of the papillae. Cilia clusters were identified anatomically, and spontaneous active spiking was recorded. Spiking discharges were stimulated by both mechanical and chemical stimuli, by changes in pressure in the perfusion recording electrode and by subsequent perfusion of the amino acid derivative betaine, an appetent found in high concentrations in invertebrates like those on which *Pleurobranchaea* preys (cf. [6]). Correspondingly, in intact animals appetitive chemical stimuli sum with mild mechanical stimulation to initiate feeding responses [14]. It may be that mechano- and chemosensitivity are segregated to different cells of the cluster, but it seems likely that the sensory cells mediate both mechanical and chemical information like mammalian olfactory cells [15]. Responsiveness to appetitive stimulation by the afferents may thus encode information to send to the feeding motor network, as well as to the turning motor network for targeting stimuli.

It is likely that the ciliated receptor cells transmit sensory information synaptically to secondary afferents in the sensory nerves. We could not directly observe the action potentials of the primary receptor cells in the sensory nerves. While it is possible that their axons are too small for their spike currents to be easily recorded with conventional suction electrodes, much like the minute C fibers of vertebrate sensory nerve, they should have emerged in the averaging of multiple stimulus records (Fig 10C). Moreover, antidromic spikes could not be recorded in papillae by stimulation of the sensory nerve. Finally, in no single one of our histological observations were axons seen to clearly originate from the ciliated cells. Thus, it is most likely that the primary receptors of the cilia clusters synaptically drive the relatively few recordable secondary afferents in the sensory nerves.

It was of marked interest to observe the SeN of putatively dopaminergic neural processes just below the cilia clusters, which appears to connect the clusters with each other and to the sensory nerves. It is markedly similar to the TH-li plexus described for the peripheral sensory network of the siphon of *Aplysia*, which was suggested to constitute a self-contained sensorimotor system for peripheral detection of, processing of, and responses to mechanical stimuli [16]. The meshwork likely represents elements of the neural network responsible for the averaging of multiple stimulated loci on the oral veil, likely including other neurotransmitters like GABA, ACh, glutamate and peptides. Yafremava and Gillette [2] recorded the axons in the sensory nerves to find that these neurons showed place coding in their population responses for distinct stimulus sites along the oral veil and that their activity averaged multiple stimulus sites. Those observations led to the predictions that recurrent inhibitory networks mediating place coding occur in the periphery.

It was also of interest to find that the putatively dopaminergic ciliated sensory cells represented a significant minority, perhaps 10%, of the cells in the clusters. In gastropods dopaminergic and histaminergic putative primary sensory receptors are plentiful in cephalic chemotactile sensory areas [3,16–22]. Moreover, other transmitters potentially present are glutamate, GABA, serotonin, and peptides, and it is not yet known which are involved in stimulation of the feeding and/or turn networks. The potential roles of the dopaminergic receptors and secondary afferents in mediating appetitive stimulation bring up the questions whether DA contributes to sensory processing by metabotropic receptors, as suggested by the effects of sulpiride [3], and whether DA might affect the odor signal averaging of the OV encoding turn amplitudes [1,2] (also cf. [23]). Miller [24] has pointed out how the comparative anatomy and physiology of dopamine localization and function in gastropods has important evolutionary implications.

A curious observation was the possible presence of paddle cilia inside some of the small tubercles in the skin surrounding the cilia clusters, for which slight mechanical damage in SEM processing broke the surfaces of some tubercles to reveal them. They are unlikely to have a normal chemosensory function as they are isolated from the exterior under the skin. Potentially, those hidden putative cilia correspond to the sparse labeling seen with anti-tubulin staining scattered outside of the clusters. As they did not show distinct afferent axons, their actions might be locally paracrine and possibly involved in the defensive skin acid secretion induced by rough mechanical and chemical stimulation [11].

Other clues to PNS network function may arise from previous descriptions of serotonergic innervation of the oral veil PNS by identified cells in the cerebral lobes of the central cerebropleural ganglia, the C-cluster [25,26], which were earlier found to be synaptically coupled to the feeding motor network [27]. These observations are consistent with the spikes recorded in the papillae on stimulation of the LOVN (Fig 12) being carried by a serotonergic efferent axon. A possible peripheral neuromodulatory function of the serotonergic connections is to regulate the gain of the sensory cells. This would couple the function of the feeding network in expressing the appetitive state of the animal to regulating sensory sensitivity to chemotactile stimuli (cf. [7,28]).

**Fig 13:**
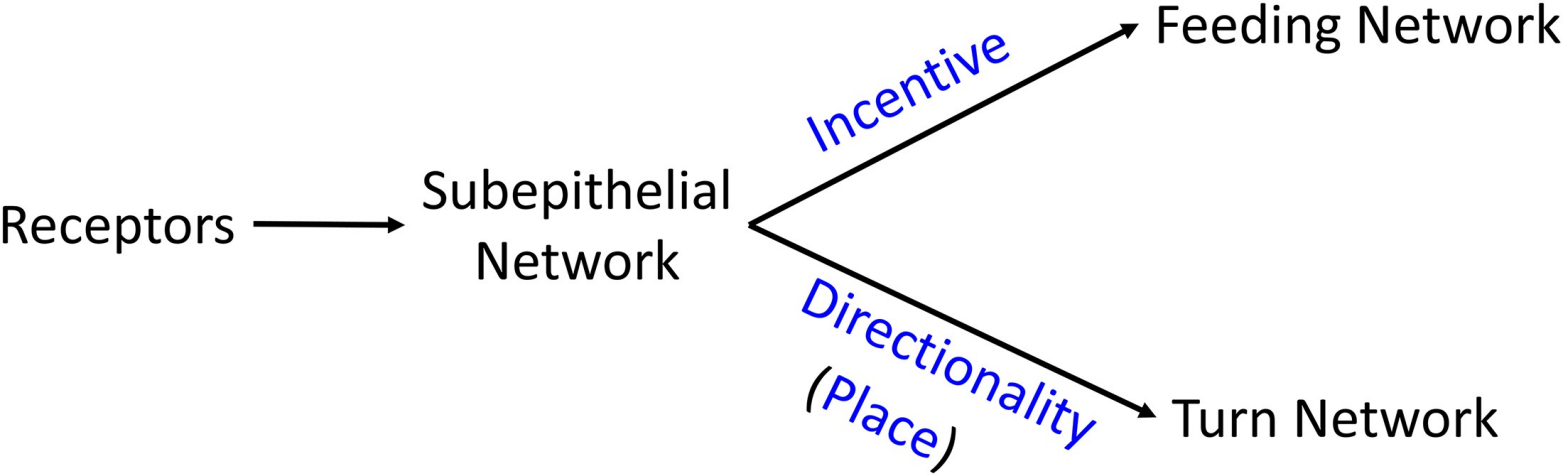
The flow of sensory information through the SeN for stimulus incentivization and localization. A simple model summarizing the inferred actions of the SeN in encoding and mediating information on appetitive sensory qualities and location for approach-avoidance decisions.

The dual functions of the SeN in mediating appetitive incentive to the feeding motor network and place code for stimuli sources with respect to the oral veil are summarized in Fig 13. These resemble in lesser detail those of the vertebrate pallial forebrain, whose dual major functions are also stimulus incentivization and localization for decisive, targeted action selection in terms of posture, locomotion, and motor arousal [29]. In its marked dopaminergic nature, the SeN resembles the mammalian olfactory bulb, where interestingly the neuromodulatory effects of the dopaminergic neurons are critical to olfactory function (review: [30]).

These observations indicate potential for marked complexity in network organization and sensory integration in the PNSs of soft-bodied animals. Previously, such complexity has been better appreciated in the image forming eyes of some pulmonate gastropods that may discriminate among visual objects in the environment (review: [31]), in the simpler eyes of nudibranchs like *Hermissenda* with interestingly complex integrative functions (review: [32]), and in the tactile and visual circuits of the cephalopods (reviews: [33,34]). Gastropods and cephalopods, as sister clades, share many nervous system similarities, and it may be expected that further similarities are to be found in the sensory functions and circuitries of both their central and peripheral nervous systems.

Potential clues to the nature of the sensory afferents that carry chemotactile information from the SeN come from previous identification of gastropod sensory neurons. Bicker et al. [35,36] identified multimodal sensory interneurons in peripheral ganglia of rhinophores and tentacles of *Pleurobranchaea* to show that they integrated sensory input for relay to the CNS. In the nudibranch *Tritonia diomedea*, cell bodies of similar multimodal neurons in the cerebral ganglion lobes were found to innervate the cephalic oral veil, which lacks the larger peripheral ganglia of rhinophore and tentacle [37,38]. Multimodal sensory neurons with cell bodies in tentacle ganglia and CNS cerebral lobes thus seem likely to carry directional information from the SeN to the network mediating posture and locomotion [39].

Finally, the observations of a SeN likely to mediate place coding as a motor template for the turn premotor network [40] may be speculatively interpreted in the context of the evolution of place coding central brain structures in vertebrates and arthropods. Tomer et al. [41] and others (cf. [42]) have provided evidence consistent with a common origin of arthropod and annelid mushroom bodies and vertebrate pallium from an ancestral olfactory network: that they develop from similar molecular coordinates in a similar molecular brain topology, that their development involves conserved patterning mechanisms, and that they display neuron types that must have already existed in the protostome-deuterostome ancestors. The inferred deep homology of pallium and mushroom bodies suggest that they were present in the common ancestral bilaterian before the evolution of exo- and endoskeletons, segmentation, and articulated appendages. The computational networks for place coding in molluskan chemosensory structures like the SeN of *Pleurobranchaea*’s oral veil provide candidate neuronal network types that could have been adapted to spatially mapping the environment and incorporated centrally in evolution.

The PNSs of the oral veil of *Pleurobranchaea* and other gastropods are open to future analyses of sensory computations. So far, those computations are found to encode more than the simple magnitudes of chemotactile stimuli. They also compute place codes for the most likely direction of a stimulus source through averaging multiple simultaneous stimulation sites on the oral veil [2]. In the CNS, these signals must act in dual roles, first as incentivizing stimuli at the feeding network, and second as directional templates for motor responses that can function either to target or to avoid the most likely stimulus source direction. Thus, these observations add further background to investigating PNS circuitry for computations in the connections between the primary sensory cells and the larger interneurons of the sensory nerves. Three addressable questions are whether the sensory signatures for chemical stimuli can be discriminated in the electrical activity, whether memory for trained odor discrimination might be stored in the PNS, and the roles of the serotonergic innervation in the SeN for attention and memory?

## Supporting information

S1 Fig (TIF)
