# Correction: A peripheral subepithelial network for chemotactile processing in the predatory sea slug *Pleurobranchaea californica*

**DOI:** 10.1371/journal.pone.0319277

**Published:** 2025-02-10

**Authors:** Tigran Norekian, Yichen Liu, Ekaterina D. Gribkova, Jilai Cui, Rhanor Gillette

There are errors in the Funding statement. The correct Funding statement is as follows: This work is supported by Office of Naval Research Multidisciplinary University Research Initiatives Program grant N00014-19-1-237 to RG. TN is also supported in part by National Science Foundation grant 1557923 and National Institute of Neurological Disorders and Stroke grant R01NS114491 to LL Moroz. The funders have no role in study design, data collection and analysis, decision to publish, or preparation of the manuscript.

Fig 6 is missing the scale bars. Please see the correct Fig 6 here.

**Fig 6:**
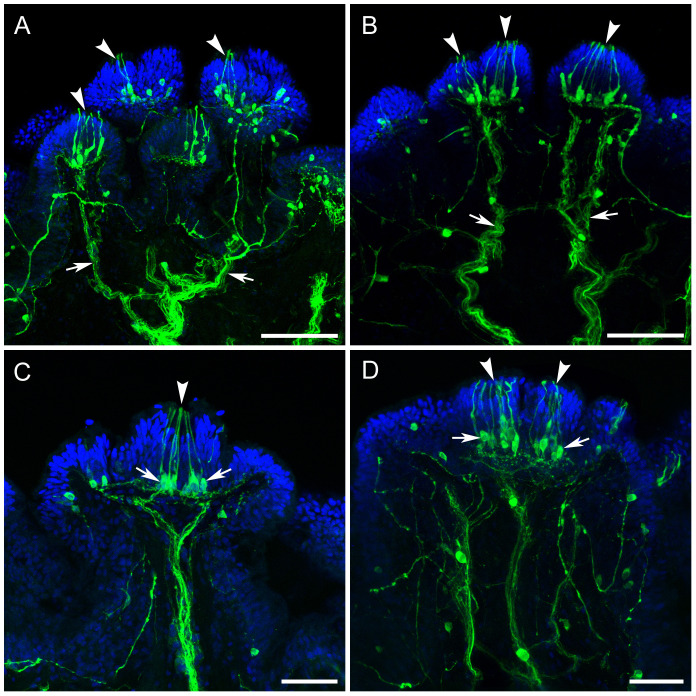
TH-li in branching nerves and sensory clusters. TH-li staining of cilia clusters and nerves is green; DAPI staining of cell nuclei in the epithelium is blue. **A, B**: Thick branching TH-li nerves (arrows) approach the clusters of cilia (arrowheads) described earlier. Note individual TH-li neuronal cell bodies spread around nerve branches and lateral branchlets near cilia clusters’ bases. **C, D**: Several TH-li cell bodies (arrows) each produces a single cilia-like projection to the surface. These presumed receptor cells lie at the base of the cilia cluster (arrowhead). Neuropil-like areas are visible below clusters. An axon of a cell body of these TH-li cells at the left arrow in C branches laterally. Scale bars: A and B, 100 μm; C and D, 50 μm.

## References

[1] Norekian T, Liu Y, Gribkova ED, Cui J, Gillette R (2024) A peripheral subepithelial network for chemotactile processing in the predatory sea slug *Pleurobranchaea californica*. PLoS ONE 19(2): e0296872. 10.1371/journal.pone.0296872 38329975 PMC10852322

