# Supplementary figures and images for: A peripheral subepithelial network for chemotactile processing in the predatory sea slug Pleurobranchaea californica
Source: PLoS One. 2024 Feb 8;19(2):e0296872. doi: 10.1371/journal.pone.0296872 (PMC10852322; doi:10.1371/journal.pone.0296872)

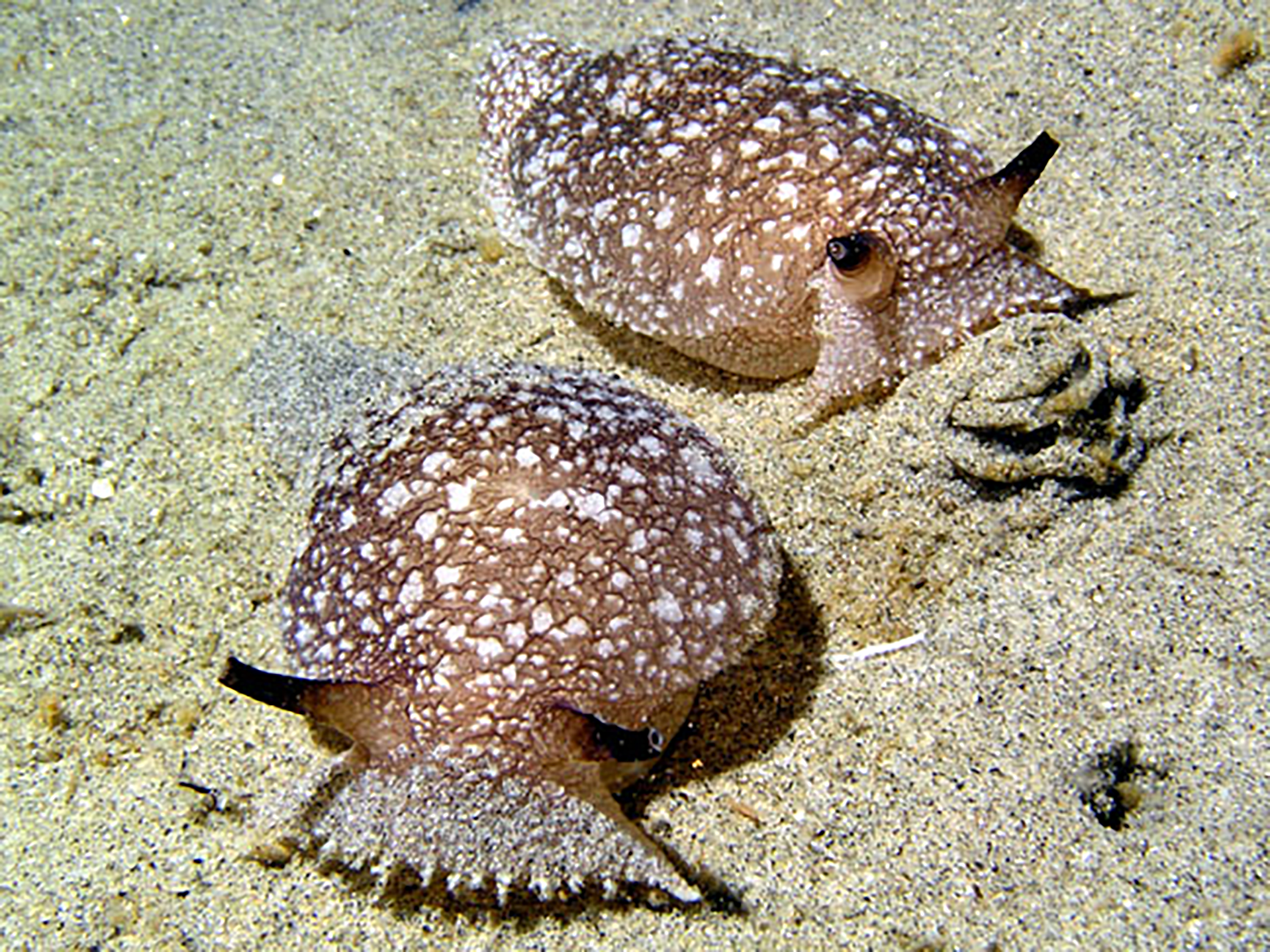

Supplement: S1 Fig — (TIF) [file pone.0296872.s001.tif]
